# Multifunctional PLGA/collagen/zeolitic imidazolate framework-8 composite nanofibrous membranes for guided bone regeneration

**DOI:** 10.3389/fbioe.2025.1611948

**Published:** 2025-06-27

**Authors:** Tianqi Wang, Qi Xie, Hongbo Liang, Yu Sun, Weili Xie

**Affiliations:** ^1^The First Affiliated Hospital of Harbin Medical University, School of Stomatology, Harbin Medical University, Harbin, China; ^2^ School of Materials Science and Engineering, Harbin Institute of Technology, Harbin, China

**Keywords:** electrospinning, nanofibrous membrane, ZIF-8, tensile strength, guided bone regeneration

## Abstract

**Introduction:**

Guided bone regeneration (GBR) is widely used for maxillofacial defects, but fabricating membranes that enhance osteoinduction and antimicrobial resistance remains challenging. This study addresses critical bone defect therapy by developing collagen (Col) and zeolitic imidazolate framework-8 (ZIF-8) reinforced poly(lactide-co-glycolide) (PLGA) nanofibrous membranes.

**Methods:**

Material characterization analyzed the composite nanofibrous membranes’ morphology, structure, wettability, tensile strength, *in vitro* degradation, and ion release. Biocompatibility and osteogenesis were evaluated using live/dead staining, cytoskeleton staining, CCK-8 assay, alkaline phosphatase (ALP) activity quantification, ALP staining, alizarin red S (ARS) staining and ARS quantification. Antibacterial efficacy was assessed via agar plate counting and bacterial growth kinetics. *In vivo* bone regeneration was examined in a rat cranial critical defect model treated with the membrane; bone formation was evaluated by Micro-CT and hematoxylin-eosin staining after 4 weeks.

**Results:**

Optimization of the PLGA/Col weight ratio (100:3) yielded composite membranes demonstrating superior tensile strength. The PLGA/Col/ZIF-8 nanofibrous composite incorporating 1 wt% ZIF-8 nanoparticles exhibited optimal biocompatibility with sustained Zn^2+^ release kinetics. *In vitro* experiments demonstrated that sustained release of Zn^2+^ has the dual effects of stimulating osteogenic differentiation and effectively preventing early bacterial infection. *In vivo* a rat calvarial defect model further confirmed the positive bone regeneration effect of the PLGA/Col/ZIF-8 composite nanofibrous membrane.

**Discussion:**

PLGA/Col/ZIF-8 composite nanofibrous membranes have great potential for application in guiding bone tissue regeneration.

## 1 Introduction

The incidence of alveolar and maxillofacial bone defects caused by trauma, tumor and inflammation is high, and such pathological bone defects often lead to biomechanical stability and functional reconstruction of conventional restorations and implant-supported prosthetic solutions (Visscher et al., 2016; Yang et al., 2022). In this context, revolutionary advances in bone tissue engineering technologies are providing breakthrough solutions for the regenerative repair of bone defects. Guided bone regeneration (GBR) biomaterials can significantly improve the effectiveness of structural and functional reconstruction of critical-sized bone defects (Urban and Monje, 2019). The core principle of GBR is to create a physical barrier between the bone defect area and the soft tissue by using a biological barrier membrane that selectively inhibits the infiltration of fibrous connective tissue and epithelial cells based on a spatial competition mechanism, while creating a preferential microenvironment for the proliferation of osteogenic progenitor cells. The barrier membrane maintains the stability of the clot and the morphology of the defect area through mechanical isolation and relieves pressure on the soft tissues, thereby supporting the process of directed migration, differentiation and mineralization of osteoblasts (Laney, 2017; Sheikh et al., 2017).

Currently, GBR membranes are mainly divided into non-resorbable and resorbable materials. Although polytetrafluoroethylene (PTFE) is clinically effective as a non-resorbable membrane, its non-resorbability requires secondary surgical removal, which significantly increases the risk of postoperative infection and limits its clinical use (Carbonell et al., 2014; Garcia et al., 2018; Trobos et al., 2018). In contrast, resorbable collagen (Col) membranes, with their excellent biocompatibility, biodegradability and natural bioactivity, have become an important alternative in bone augmentation therapy, promoting bone repair through tissue regeneration mechanisms while avoiding secondary surgery (Dimitriou et al., 2012; Liu and Kerns, 2014). However, the poor mechanical properties and rapid degradation behavior of collagen membranes have hindered their development, as they are unable to maintain their spatial configuration (Garcia et al., 2017; Rothamel et al., 2005). Micro- and nanoscale fiber structures produced by electrospinning technology have been widely used in the development of GBR membranes due to their highly biomimetic properties. These structures can mimic the topological characteristics of the natural extracellular matrix (ECM) and effectively promote cell adhesion, proliferation and metabolic exchange, while the micro-barrier formed by the nanofiber network can selectively block the invasion of fibrous connective tissue and ensure the stability of the osteogenic microenvironment in the bone defect region. However, these electrostatically spun GBR membranes have not yet been clinically translated (Martins et al., 2020; Wang et al., 2019; Wang et al., 2013). Therefore, the development of GBR membranes with good mechanical properties, degradation rate, osteoinductive activity and antimicrobial functionality required to withstand the multidimensional effects of mechanical stress, microbial colonization and biomolecular metabolism in the dynamic oral cavity bioenvironment is a core research direction.

Col, a core component of connective tissue, is widely used in biomaterial design due to its low immunogenicity, biodegradability and structural support function (Ren et al., 2022). Poly (lactide-co-glycolide) (PLGA), an FDA-approved biodegradable polyester, has proven to be the preferred substrate for bone tissue engineering scaffolds due to its combination of excellent mechanical strength and histocompatibility (Brown et al., 2018). Based on this, this study aims to improve the interfacial compatibility and mechanical stability of electrostatically spun fiber membranes through intermolecular interactions by the composite of Col and PLGA (Ahi et al., 2020; Kwak et al., 2016; Yang et al., 2018). Prior research shows that PLGA/Col composite systems have been validated in the field of nerve axon guidance scaffolds, brain injury repair carriers and bone defect filling materials (Park et al., 2018). However, the composite system still suffers from significant functional deficiencies in terms of osteoinductive differentiation efficiency and antimicrobial activity in guided bone regeneration membrane applications.

The dual role of bioactive ions in tissue repair and bacterial resistance modulation is gradually becoming a research focus. Zinc, an essential trace element, plays a key biological function by regulating osteoblast differentiation, matrix mineralization and maintenance of bone homeostasis (Yamaguchi and Weitzmann, 2011). In addition, its antimicrobial activity has been widely demonstrated (Nagata and Lonnerdal, 2011). However, excessive zinc ions have the potential to cause toxicity and to inhibit the expression of bone-related genes. Therefore, a controlled slow-release system is required to achieve zinc ion delivery (Giliopoulos et al., 2020; Yang and Yang, 2020). Metal-organic frameworks (MOFs) are a class of porous materials self-assembled by coordination between organic ligands and metal ions, among which zeolite imidazolate framework-8 (ZIF-8) is composed of zinc ions coordinated with dimethylimidazole, which combines excellent biocompatibility and osteoinductive properties (Pan et al., 2011). The material exhibits stability in neutral environments, yet rapidly dissociates under acidic conditions, thereby facilitating the prompt release of Zn^2+^ in bacterial infection microenvironments (Sun et al., 2012). It has been demonstrated that nano ZIF-8 activates the MAPK signaling pathway in bone marrow mesenchymal stem cells through the process of endocytosis, thereby significantly promoting osteogenic differentiation (Gao et al., 2021; Wang et al., 2016). Therefore, ZIF-8 nanoparticles can be introduced into GBR membranes as antimicrobial-osteogenic functional modification materials to achieve the membrane material to play a precise regulatory role of antimicrobial and osteogenic in the process of bone repair.

In this study, a novel PLGA/Col/ZIF-8 composite nanofibrous membrane was fabricated by electrospinning technology for bone defect repair. Initially, a mechanically adapted substrate was constructed by means of gradient doping Col in a PLGA matrix, followed by the subsequent integration of ZIF-8 into PLGA/Col to achieve a microenvironment-triggered release mechanism of zinc ions, thereby resulting in both antimicrobial and osteogenic effects. Furthermore, a rat cranial defect model was utilized to confirm its capacity to repair bone tissue defects *in vivo*, thereby assessing the potential of this composite fibrous membrane in bone regeneration strategies.

## 2 Materials and methods

### 2.1 Materials and reagents

Poly (lactic-co-glycolic acid) (PLGA; LA/GA = 75:25, Mw = 66–107 kDa) was procured from Sigma Aldrich. Type I collagen (Col I) was obtained from Macklin, while 1,1,1,3,3,3-hexafluoro-2-propanol (HFIP) and 2-methylimidazole (2-MIM) were sourced from Aladdin. Zinc nitrate hexahydrate was procured from Innochem. All of the aforementioned chemical reagents were of analytical grade and did not undergo any further purification.

### 2.2 Fabrication of PLGA/col/ZIF-8 electrospun membrane

The synthesis and characterization of the ZIF-8 nanoparticles were conducted in accordance with the following procedure (Supplementary Figures S1, S2). The electrospun solution was prepared by dissolving PLGA in HFIP (20% w/v) under magnetic stirring at room temperature. To prepare the PLGA/Col electrospun solution, 1 g of PLGA was dissolved in 5 mL of HFIP. Subsequently, the different weight ratios of Col/PLGA (1:100, 3:100, 5:100 and 10:100; abbreviated as PC1, PC3, PC5 and PC10, respectively) were added under stirring until complete dissolution. The electrospun solution of PLGA/Col/ZIF-8 was prepared in the following manner: ZIF-8 at varying weight ratios (0.5 wt%, 1 wt%, 1.5 wt% and 2 wt%; designated as PC3Z0.5, PC3Z1, PC3Z1.5 and PC3Z2, respectively) was subjected to sonication and dispersion in 5 mL HFIP. Subsequently, 1 g of PLGA and 0.03 g of Col I were added to the solution, which was then magnetically stirred until complete dissolution of the PLGA and Col I had occurred. The solution was then sonicated for a further 0.5 h to ensure uniform dispersion of the nano-ZIF-8 within the spinning solution. The electrospun solutions were introduced into the system via a 23 G flat-tipped needle at a constant injection rate of 0.5 mL/h, using a plastic syringe. The distance between the needle and the flat collector was 15 cm, the applied positive voltage was 10–15 kV, and the humidity was approximately 21%–31%.

### 2.3 Characterization

The microscopic morphology of the membranes was examined using a scanning electron microscope (SEM, Gemini 560, ZEISS, Germany). The chemical compositions of the membranes were established through the use of a Fourier transform infrared spectrometer (FTIR, Nicolet iS5, Thermo, United States). Moreover, Nano Measurer was employed to quantify the diameter and distribution of the membranes from the acquired SEM image. The presence of ZIF-8 crystals in the membranes was confirmed through X-ray diffraction (XRD, X’PERT, Panalytical, Holland). Thermogravimetric analysis (TG, STA449F3, NETZSCH, Germany) was employed to ascertain the thermal stability of the membranes. All samples were subjected to a heating process, increasing the temperature from 30°C to 500°C at a rate of 10°C/min, in a nitrogen-enriched environment. The wettability of the membranes (n = 3) was evaluated using a contact angle meter (SDC 350, SINDIN, China). In order to evaluate the impact of Col and ZIF-8 NPs incorporation on the mechanical properties of the membranes (n = 3), an electronic universal testing machine (Model 6103, MTS, China) with a 50 N load cell was employed to conduct a series of tests on the membranes under dry and wet condition (soaking in deionized water for 24 h), which had overall dimensions of 50 mm × 10 mm × 0.2 mm. The crosshead speed was set at 15 mm/min. For degradation test, membrane samples (n = 3) were precisely sectioned into 10 mm × 10 mm squares. Initial dry mass (W_0_) was determined using an analytical balance. Specimens were subsequently immersed in 10 mL of phosphate-buffered saline (PBS, pH 7.4) and incubated at 37°C. The PBS was replenished weekly to maintain ionic stability. The samples were removed at each predetermined time point, at days 1, 3, 7, 14, 21, 28 and 56, and then placed in a vacuum drying oven until completely dry and weighed (W_t_). Weight loss (%) = (W_0_ - W_t_)/W_0_ × 100. The release of zinc ions from the composite membranes (n = 3) was quantified using inductively coupled plasma emission spectroscopy (ICP-OES, iCAP 7,400, Thermo, United States). The membranes were immersed in PBS with a specified pH value (pH 7.4) for 1, 3, 5, and 7 days at 37°C without agitation, the extracts were collected at various time points and the amount of released Zn^2+^ ions was measured.

### 2.4 Biocompatibility *in vitro*

#### 2.4.1 Cell culture

The mouse bone marrow mesenchymal stem cells (BMSCs) were procured from Immocell Biotechnology. The BMSCs were cultured in Dulbecco’s Modified Eagle Medium: F-12 (DMEM/F12, Pricella, China) supplemented with 10% Fetal Bovine Serum (FBS, Gibco, United States) and 1% mixture of penicillin-streptomycin (Gibco, United States), and passaged every 2 days. The osteogenic differentiation of BMSCs was induced using an osteogenic induction medium (OriCell, China). All cells were cultured in accordance with standard conditions (37°C, 5% CO_2_, 95% relative humidity). The fiber membranes employed in all cell experiments were subjected to a pre-processing step involving immersion in a 75% ethanol solution for 1 hour. Thereafter, the prepared membranes underwent a sterilization procedure involving exposure to ultraviolet light.

#### 2.4.2 Cell proliferation

BMSCs were seeded on the fiber membranes (n = 4) in 96-well plates at a cell density of 2.5 × 10^3^ cells per well. Following a period of cultivation lasting for 1, 3, and 5 days, the extent of cellular proliferation was gauged through the utilization of the cell counting kit-8(CCK-8, MedChemExpress, China). At the designated time point, the cells were rinsed with PBS and cultured in a medium containing 10% CCK-8 solution at 37°C for 2 h. Subsequently, the optical density (OD) value at 450 nm was determined using a microplate reader.

#### 2.4.3 Cell live/dead staining

The cells were seeded on the fiber membranes (n = 4) in 24-well plates at a density of 2 × 10^4^ cells per well. Following a 24 h incubation period, the working solution was prepared at a ratio of 1:1:1,000, comprising Calcein AM, PI and detection buffer. Subsequently, the solution was added to each well and incubated at 37°C for 30 min in the dark. The presence of live (green) and dead (red) cells were observed using fluorescence microscopy.

#### 2.4.4 Cell morphology

The cells were seeded on the membranes (n = 4) in 24-well plates at a density of 2 × 10^4^ cells per well for a 24 h culture period. Subsequently, the cells were fixed with 4% paraformaldehyde and rinsed with PBS. They were then permeabilized with 0.5% Triton X-100 and rinsed with PBS. Subsequently, F-actin (red) and nuclei (blue) were stained with TRITC-phalloidin and 4, 6-diamidino-2-phenylindole dihydrochloride (DAPI) in the dark, respectively, and observed using fluorescence microscopy.

### 2.5 Osteogenic ability *in vitro*

#### 2.5.1 ALP activity

BMSCs were seeded at a density of 2 × 10^4^ cells per well on the membranes (n = 4) in 24-well plates. The osteogenic induction medium was replenished every 2 days. Following a 7-day and 14-day cultivation period, a quantitative evaluation of ALP activity was conducted in accordance with the instructions provided in the ALP detection kit (Beyotime, China). For qualitative analysis of ALP, BMSCs were fixed with 4% paraformaldehyde for 15 min at room temperature and subsequently stained with the BCIP/NBT ALP kit for 30 min in the absence of light.

#### 2.5.2 Alizarin red S staining

BMSCs were seeded at a density of 2 × 10^4^ cells per well on the membranes (n = 4) in 24-well plates. Following a 21-day period of cultivation in an osteogenic induction medium, the extracellular matrix mineralization capacity was evaluated through the use of ARS staining. The BMSCs were fixed with 4% paraformaldehyde for a period of 30 min, after which they were stained with alizarin red S (OriCell, China) for a further 10 min at 37°C. The stained samples were thoroughly washed with PBS to remove any residual dye, and images were acquired under a stereomicroscope. 10% cetylpyridinium chloride was added to each well, and the stained mineralized nodules were solubilized by shaking the bed for 30 min. The absorbance at 630 nm was then detected by a microplate reader.

### 2.6 Antibacterial assay

#### 2.6.1 Bacterial culture

*Escherichia coli* (*E. coli*) and *Staphylococcus aureus* (*S. aureus*) were retrieved from the −80°C refrigerator, with individual colonies selected and dispersed in medium. The suspension was then shaken and cultured at 37°C with 130 rpm agitation, resulting in a bacterial concentration of 1 × 10^6^ CFU/mL, which was subsequently utilized in subsequent experiments.

#### 2.6.2 Agar plate count

At 37°C, samples (n = 3) were cocultured with *E. coli* and *S. aureus* for 24 h. Thereafter, 60 μL of broth was aspirated and diluted to specific concentrations as needed. Subsequently, 100 μL of the diluted broth was spread evenly on solid agar plates. After 12 h of continuous incubation at 37°C, the number of colonies on the plates was counted and photographed.

#### 2.6.3 Bacterial growth curve

The three groups of nanofiber membranes (n = 3) were co-cultured with *E. coli* or *S. aureus* and placed in a bacterial shaking incubator at 37°C. At specific time points (0, 2, 4, 6, 8, 10, and 12 h), an appropriate volume of bacterial suspension was aspirated, transferred to a 96-well plate, and the OD at 600 nm was measured using a microplate reader. Bacterial growth curves were then plotted based on the OD values.

### 2.7 Animal experiments

#### 2.7.1 Animal grouping and surgery

All animal procedures were approved by the Ethical Committee on Animal Experiments of Harbin Medical University. Twenty male Sprague-Dawley rats were randomly divided into four groups (n = 5): blank control group, P, PC, and PCZ. Under general anesthesia, the dorsal area was shaved and disinfected, followed by the creation of a 2 cm skin incision to form a subcutaneous pocket. After implantation of the corresponding materials, the wound was closed using non-absorbable monofilament sutures. Peripheral blood samples were collected 3 days post-surgery for biochemical analysis.

Twenty Sprague Dawley rats were anaesthetized, the surgical site on the top of the head was prepared and disinfected, and a longitudinal incision was made in the middle of the skull, with the periosteum elevated to expose the cranial surface. A circular bone defect measuring 5 mm in diameter was created on the side of the middle cranial suture using a ring bone extraction drill. The bone defect was full-length and did not damage the dura mater. During the procedure, saline rinsing of the ring drill was ensured for cooling protection. Twenty Sprague-Dawley rats were randomized into four groups (Blank; P; PC; PCZ). Calvarial defects in three treatment groups (n = 5) received membrane implantation, while the blank control group remained non-interventional. Following the implantation of the membrane material, the subcutaneous tissue was closed and the skin layer incision was sutured. The rats were euthanized 4 weeks after the surgical procedure. The removal and fixation of the cranial tissues, as well as the heart, liver, spleen, lung and kidney organs, was conducted using 4% paraformaldehyde for subsequent analysis.

#### 2.7.2 Blood biochemistry assay

Blood samples were collected for the purpose of quantifying various hematological parameters, including white blood cells (WBC), red blood cells (RBC), hemoglobin (HGB), platelets (PLT), aspartate aminotransferase (AST), alanine aminotransferase (ALT), blood urea nitrogen (UREA) and creatinine (CREA).

#### 2.7.3 Micro-CT analysis

Micro-CT was utilised to scan the fixed cranial tissue blocks, thus facilitating analysis of bone regeneration at the defect site in each group. The cranial scanning parameters comprised a voltage of 70 kV, a current of 114 μA, a voxel of 10 μm and a 0.5 mm aluminium filter. The 3D images were reconstructed by software, after which the bone volume fraction (bone volume/total volume, BV/TV) was measured and analysed.

#### 2.7.4 Histology staining

Following micro-CT scanning, the samples were subjected to decalcification using a 10% EDTA solution. Thereafter, they underwent a process of gradient dehydration, followed by paraffin embedding, sectioning (4 μm thickness), staining with hematoxylin and eosin (H&E), and imaging under a light microscope. For the purpose of visceral histological assessment, heart, liver, spleen, lung and kidney tissues were collected from each group and fixed in 4% paraformaldehyde. The tissues were then dehydrated in ethanol and embedded in paraffin. The tissues were sectioned into 4 μm and stained for H&E.

### 2.8 Statistical analysis

All experiments were repeated at least three times, and the data were calculated as the mean ± standard deviation. Statistical analysis of the date was performed using one-way ANOVA (GraphPad Prism 8, United States). The level of statistical significance of differences was set *P* < 0.05.

## 3 Results and discussion

### 3.1 Optimization of col and ZIF-8 content

In order to prepare PCZ composite fibrous membranes with both excellent mechanical properties and good cytocompatibility, different contents of Col and ZIF-8 were added to the PLGA fibrous membranes (the optimization process is illustrated in Supplementary Figures S3, S4, and the PCZ composite fibrous membranes with the optimal performance were selected for subsequent analysis.

**FIGURE 1:**
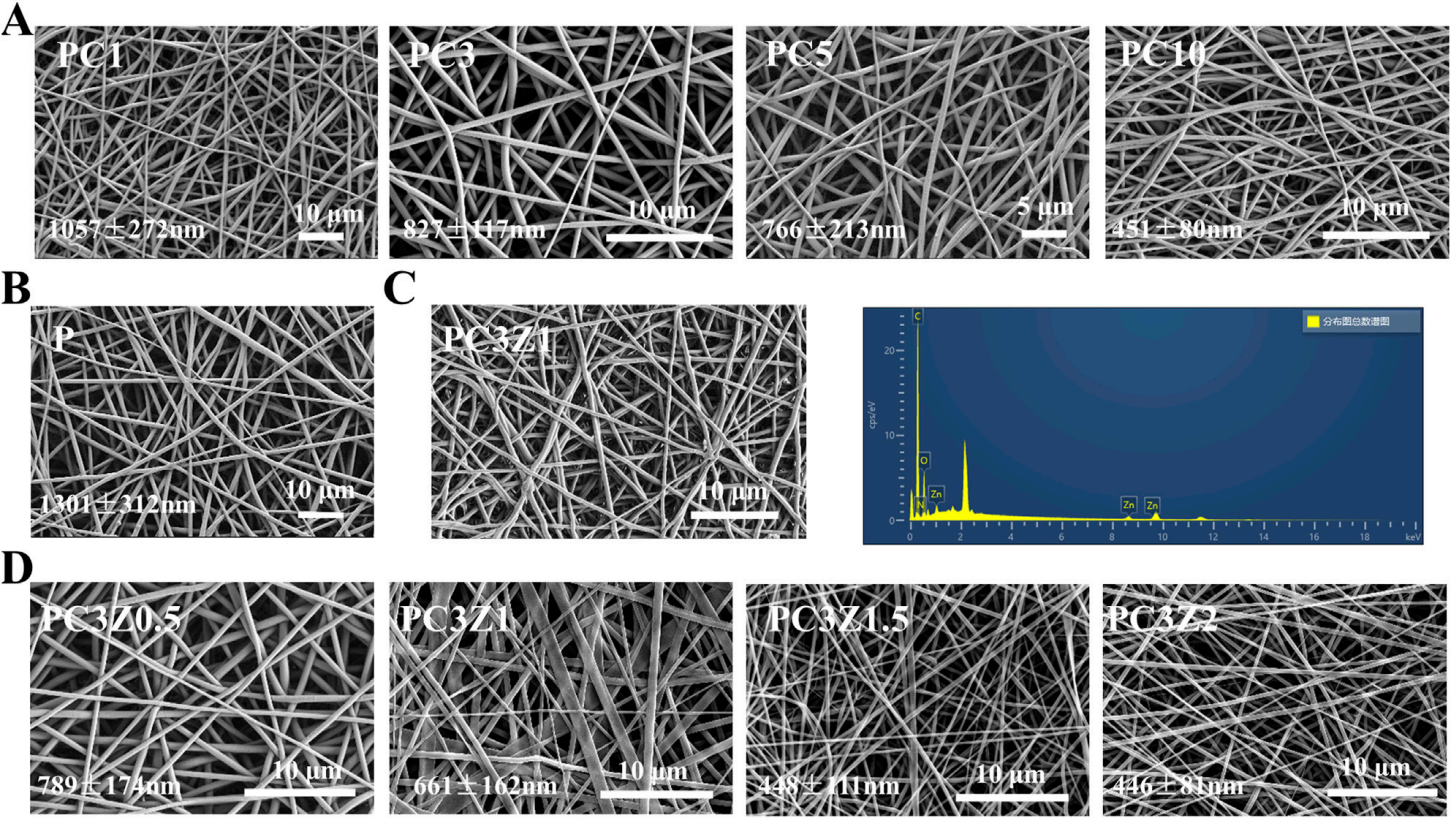
SEM images and corresponding diameter of membranes with different Col contents **(A,B)** and different ZIF-8 contents **(D)**. **(C)** Corresponding EDS spectrogram of PC3Z1 fibers.

The morphology of the membrane is illustrated in Figures 1A,B,D, where the fiber exhibit random orientation and form an interconnected porous structure. The P membranes exhibited an average fiber diameter of 1,301 ± 312 nm, demonstrating homogeneous morphological characteristics with smooth surfaces devoid of bead-like or spindle-shaped structural defects. Increasing Col content from 1% to 15% induced a progressive fiber diameter reduction from 1,057 ± 272 nm to 451 ± 80 nm in PC membranes. This inverse correlation originated from collagen’s viscosity-lowering effect on the electrospinning solution, thereby decreasing the mean fiber dimensions (Lee et al., 2009). Supplementary Figure S3 demonstrates a biphasic tensile strength response to elevated Col concentrations in the membranes. The PC3 formulation, corresponding to the tensile strength maximum, was subjected to subsequent ZIF-8 parametric analysis to optimize mechanical performance. A moderate increase in Col content can enhance the tensile properties of the material by optimizing the fiber network structure through intermolecular cross-linking; beyond the critical concentration, excess collagen triggers phase separation or crystallization defects, leading to stress concentration and reduced mechanical strength.

To determine the optimal ZIF-8 NPs loading, PC3 fibers were modified with four ZIF-8 NPs weight fractions (0.5-2 wt%). Supplementary Figure S4 demonstrated maximal proliferative activity at 1 wt% ZIF-8 on days 3/5, establishing this concentration for PCZ membrane fabrication. As shown in Figure 1D, the doping of ZIF-8 NPs led to a further decrease in the average diameter of fibers in the PCZ membrane. This originated from ZIF-8-induced viscosity reduction in the spinning solution. The initial 10 kV voltage insufficiently countered surface tension, causing droplet ejection, necessitating voltage elevation to 15 kV for stabilized fiber formation. Furthermore, EDS analysis demonstrated the presence of C, N, O and Zn, thereby further indicating the presence of ZIF-8 NPs in the nanofibers (Figure 1C). Therefore, Pure PLGA (P), PC3 (PC), and PC3Z1 (PCZ) membranes were prioritized for detailed investigation in this study.

### 3.2 Characterizations of the composite fibrous membranes

**FIGURE 2:**
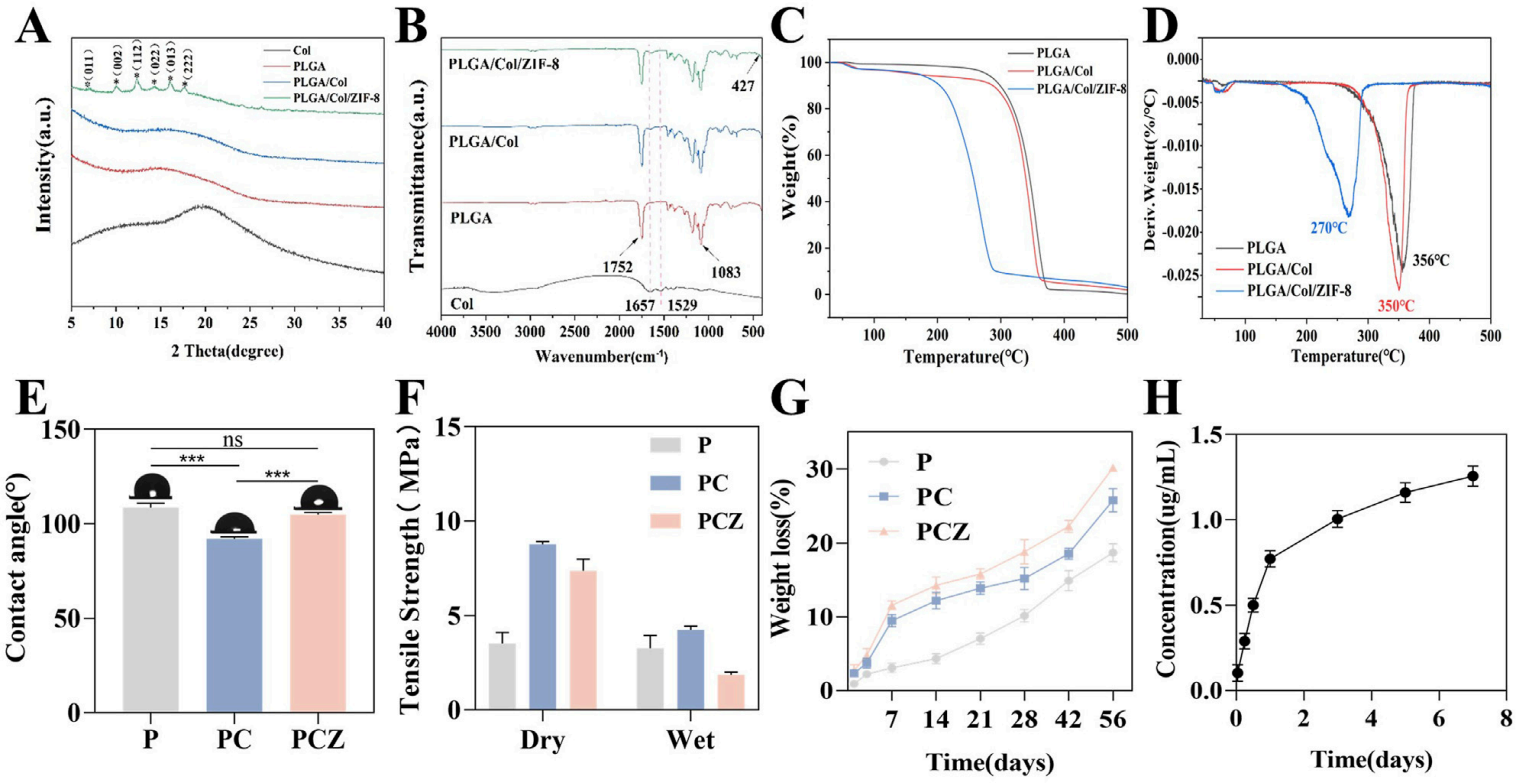
Characterization of fibrous membrane. **(A)** XRD patterns of the nanofibers. **(B)** FTIR spectra of the nanofibers. **(C)** TG analysis of the nanofibers. **(D)** DTG curve of the nanofibers. **(E)** Water contact angles of the nanofibers. **(F)** Tensile strength of the nanofibers in the dry and wet conditions. **(G)** Degradation behavior of the nanofibers after soaking in PBS solution. **(H)** Cumulative Zn^2+^ release from the PCZ nanofibers. Data are presented as mean ± SD (n = 3, *P < 0.05, **P < 0.01, ***P < 0.001).

As shown in Figure 2A, the XRD analysis of the fibrous membrane revealed that pure PLGA and Col belong to the amorphous structure, presenting only one broad peak. The incorporation of Col resulted in the diffusion and broadening of the peaks corresponding to PC and PCZ. This phenomenon may be attributed to the interaction between PLGA and Col molecules, which impedes the arrangement of PLGA chains. The XRD pattern of PCZ exhibited distinctive peaks at 7.07°, 10.03°, 12.30°, 14.32°, 16.01° and 17.64°. These peaks correspond to the ZIF-8 crystal face at (011), (002), (112), (022), (013) and (222), which indicates the presence of ZIF-8 dispersion within the nanofibers. The FTIR spectra (Figure 2B) revealed that the PLGA, PLGA/Col, and PLGA/Col/ZIF-8 exhibited stretching bands of C=O and C-O at 1752 cm^−1^ and 1,083 cm^−1^, respectively, which corresponded to the characteristic peaks of PLGA. The absorption peaks of am-ide I and amide II in Col were observed at 1,657 cm^−1^ and 1,529 cm^−1^, respectively. The absorption peaks of PLGA/Col and PLGA/Col/ZIF-8 exhibited slight shifts towards lower wavelengths, appearing at 1,644 cm^−1^ and 1,526 cm^−1^, respectively. This suggests that the amino group in Col most likely formed hydrogen bonds with the PLGA molecular chain. Additionally, PLGA/Col/ZIF-8 displayed a Zn-N stretching vibration at 427 cm^−1^.

The thermal analysis of the fibrous membrane is illustrated in Figures 2C,D. The TG curve with a first-order derivative demonstrates that the fastest thermal decomposition temperature of PLGA is 356°C, while the fastest thermal decomposition temperatures of Col and ZIF-8 are 350°C and 270°C, respectively. It can be concluded that the introduction of Col and ZIF-8 resulted in a reduction of the fastest thermal decomposition temperature of the membranes, while still meeting the thermal stability requirements of GBR membranes. The initial stage of mass loss can be attributed to the evaporation of physically adsorbed water, while the subsequent stage may be attributed to the collapse of the molecular chain structure and thermal decomposition. As displayed in Figure 2E, the PLGA membrane exhibited hydrophobic behavior, with a water contact angle of 100.43°. The incorporation of Col markedly enhanced the hydrophilicity of the membrane, reducing the surface contact angle of the material to 90.97°. This was predominantly attributed to the hydrophilic nature of the amino and carboxyl functional groups of Col. The contact angle of PCZ was 104.27°.

The mechanical strength of a material is also an important performance indicator. Accordingly, the objective of this experiment was to evaluate the mechanical properties of various membranes through a tensile strength test. As demonstrated in Figure 2F, the tensile strength of the pure P membrane in the dry state was found to be 3.52 ± 0.58 MPa. In contrast, the tensile strength of the PC composite membrane and the PCZ composite membrane was significantly higher, reaching approximately 2.5 and 2 times that of the pure P membrane, respectively. It is important to acknowledge the considerable impact of ambient humidity on the mechanical properties of materials. While the pure P membrane exhibited only 7% strength attenuation under wet conditions, the tensile strength of the PC and PCZ composite membrane decreased sharply by 52% and 75%, respectively. Specifically, the tensile strength of the PCZ membrane was measured at 7.37 ± 0.63 MPa in the dry condition, which decreased to 1.87 ± 0.14 MPa after 24 h of water immersion. A comparison with commercially available products demonstrated that the tensile strength of the Bio-Gide resorbable barrier membrane in the dry/wet state was 4.6 ± 0.94 MPa versus 1.68 ± 0.54 MPa, respectively (Raz et al., 2019). This comparison demonstrates that the mechanical properties of PCZ membranes in the wet state are still superior to those of clinically available products, which is crucial for guided bone regeneration applications.

Studies have indicated that the diameter of electrospun fibers critically influences their mechanical properties, with reduced diameters typically accompanied by enhanced strength and reduced ductility (Stachewicz et al., 2012; Tan et al., 2005). The introduction of col significantly decreased fiber diameter (Figure 1), thereby enhancing the mechanical strength of the composites. This improvement stems from two aspects. Firstly, active groups such as amino/carboxyl groups in col molecules form intermolecular hydrogen-bonding networks with PLGA, which stabilize molecular conformations and enhance macroscopic mechanical performance. As previously mentioned, XRD and FTIR results partially support this perspective. Secondly, the incorporation of ZIF-8 nanoparticles may cause disordering of polymer chain arrangements and particle agglomeration phenomena, resulting in slightly lower tensile strength in the PCZ group compared to the PC group. Nevertheless, the overall tensile performance of PCZ membranes remains significantly superior to that of pure PLGA membranes and fully meets the mechanical strength requirements for GBR applications.

In order to evaluate the *in vitro* degradability of the prepared membranes, the mass loss during their immersion was detected. As demonstrated in Figure 2G, the mass loss rate of PCZ was marginally higher than that of PC. However, the degradation kinetic curves of these two samples exhibited analogous trends. The experimental data demonstrated that the degradation rate of the pure PLGA material was significantly lower than that of the other fibrous membranes at all the detected time nodes, thereby confirming that the addition of Col changes the material properties and significantly accelerates the degradation process of the composite fibers. This phenomenon can be attributed to the formation of continuous structural defects within the composite material due to the rapid degradation of Col. This, in turn, facilitates the penetration of hydrolysis media by increasing the contact surface between the fibrous membrane and the soaking solution. Furthermore, the progressive dissolution and release of ZIF-8 NPs from the PCZ fiber membrane occurred with the extension of the degradation time, which further led to a slightly faster degradation rate of the PCZ than that of the PC membrane. Subsequently, the release behavior of Zn^2+^ from PCZ nanofibrous membranes was evaluated. As shown in Figure 2H, under simulated physiological conditions (pH 7.4, 37°C), the Zn^2+^ release profile exhibits a typical two-stage pattern: an initial burst release occurs within the first 24 h (cumulative release: 0.77 ± 0.05 μg/mL), followed by a sustained-release phase, reaching a cumulative concentration of 1.26 ± 0.06 μg/mL by day 7. Notably, this release amount is significantly lower than the internationally recognized safe threshold range for zinc ions (0.65–16.25 ppm) (Nagata and Lönnerdal, 2011). Studies have shown that zinc-based sustained-release materials generally exhibit an initial rapid release (1–7 days) followed by exponential decay, rather than a linear accumulation trend (Xue et al., 2021). With regard to the toxicity threshold, it has been confirmed *in vitro* that ZIF-8 concentrations less than 50 μg/mL possess anti-apoptotic properties and improve cell survival (Gao et al., 2021). Importantly, the peak release level of this system at 7 days was only 2.5% of that safety threshold, further corroborating its safety advantage.

### 3.3 *In vitro* biocompatibility

**FIGURE 3:**
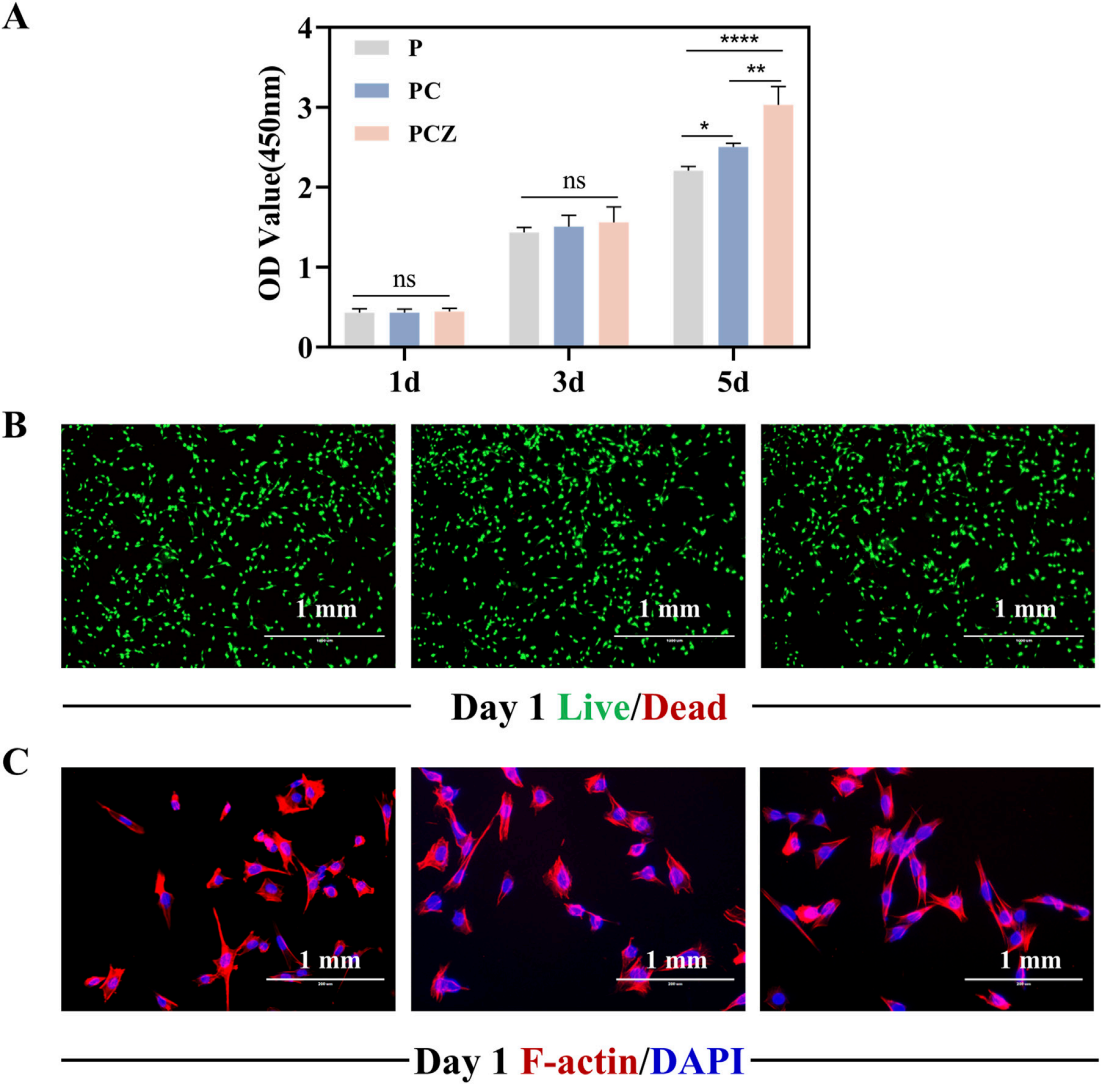
Biocompatibility of BMSCs cultured on the nanofibers *in vitro*. **(A)** Proliferation of BMSCs on the surface of the nanofibers measured by CCK-8 assay on day 1, 3, and 5. **(B)** Fluorescent staining of live/dead of BMSCs on the nanofibers at day 1. **(C)** Fluorescent staining of the cytoskeleton and nuclei of BMSCs on the nanofibers at day 1. Data are presented as mean ± SD (n = 4, *P < 0.05, **P < 0.01, ***P < 0.001).

Cell-material interactions initiate with attachment, potentially progressing to adhesion, followed by proliferation and spreading phases (Anselme, 2000). BMSCs cultured on the surface of fibrous membranes exhibited proliferation behaviors quantified via CCK-8 assay. Enhanced proliferative capacity, a cornerstone of cellular physiology, directly supports differentiation potential in biomaterial interfaces. Figure 3A indicated that the proliferation of cells on the fibrous membranes exhibited an overall increasing trend over time. There was no statistically significant difference in cell proliferation between the groups on days 1 and 3, indicating that the fibrous membranes exhibited good biocompatibility. The proliferation of BMSCs was significantly enhanced by PCZ nanofiber membranes compared to the P and PC groups on day 5. The enhanced cell proliferation capacity may be attributed to the release of zinc ions at optimal concentrations, which facilitated the proliferation of BMSCs (Yusa et al., 2011). Furthermore, cell adhesion and nutrient exchange on membranes is found to be highly correlated with fiber diameter, morphology and pore size (Zhou et al., 2015). Previous studies have shown that the number of cells adhering to nanofibers is greater than that adhering to microfibers.

Live/dead staining revealed cell viability on fibrous membranes (Figure 3B). Following 24 h culture, all groups exhibited predominantly viable cells (green) with minimal red fluorescence (dead cells), confirming the material was not significantly toxic to the cells. Given the critical role of cell adhesion in proliferation and differentiation, this study assessed the impact of fibrous membranes on the adhesion behavior and morphological spreading of BMSCs by means of a cytoskeleton-specific staining technique. Dual-channel fluorescent labeling (phalloidin for F-actin, red; DAPI for nuclei, blue) was employed (Figure 3C). After 24 h incubation, a certain number of BMSCs were observed to be adhered to the surface of the P, PC and PCZ membranes. It was evident that the incorporation of ZIF-8 NPs had a significant impact on the spreading area of the cells within the material. The cells exhibited a characteristic polygonal morphology, accompanied by filamentous pseudopod extension features, suggesting that the cells had established stable extracellular matrix interactions. It was further demonstrated that PCZ fibrous membranes would construct a growth-promoting microenvironment via strengthened cell-matrix interactions.

### 3.4 *In vitro* osteogenic differentiation

**FIGURE 4:**
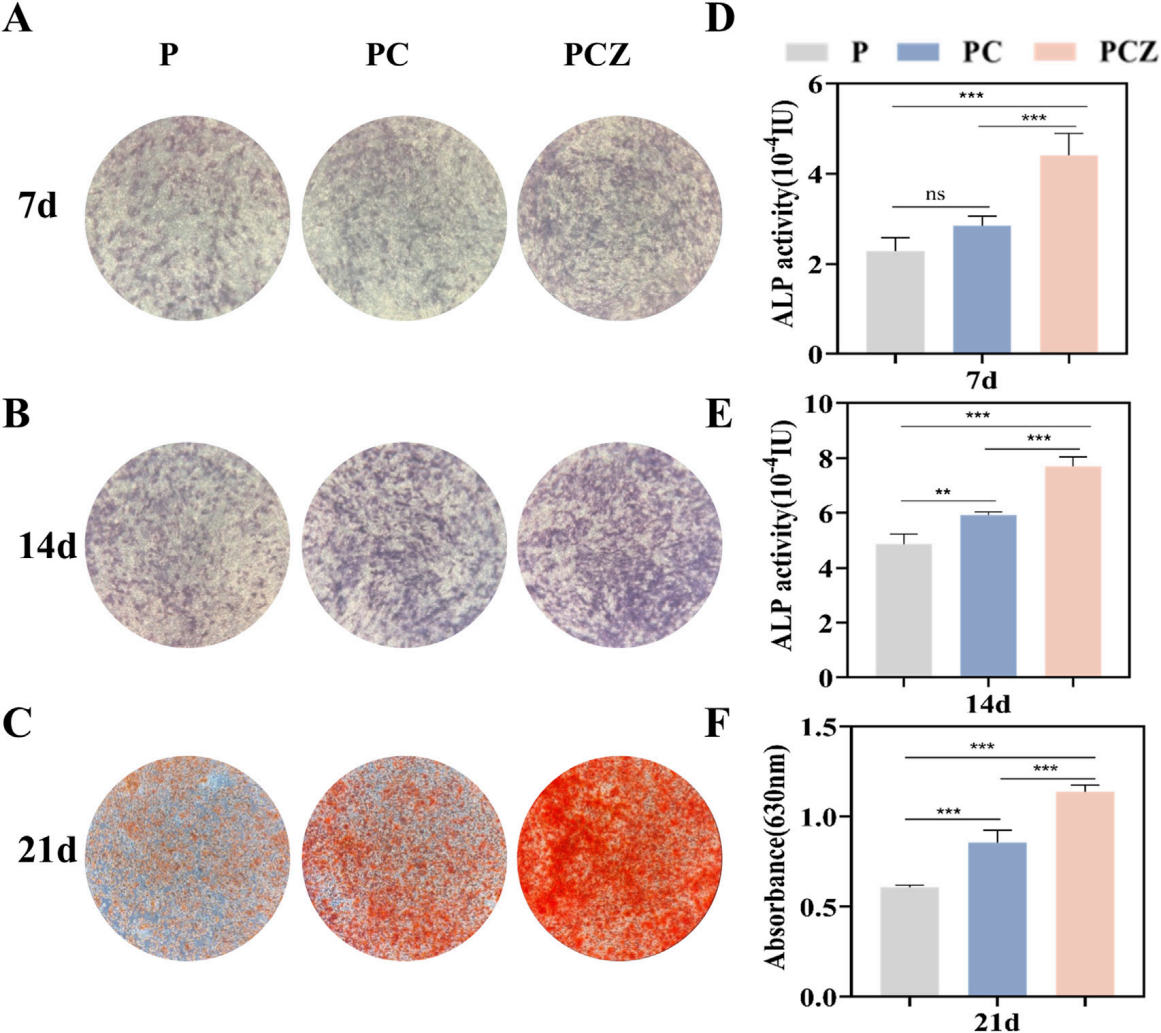
Osteogenic differentiation of BMSCs cultured on the nanofibers *in vitro*. **(A,B)** ALP staining of BMSCs cultured on the nanofibers at day 7 and 14 after osteogenic differentiation stimulation, and **(D,E)** the determination of ALP activity. **(C)** Qualitative and **(F)** quantitative characterization of Alizarin Red S staining of BMSCs cultured on different membranes at day 21. Data are presented as mean ± SD (n = 4, *P < 0.05, **P < 0.01, ***P < 0.001).

As a characteristic biomarker of early stages of osteogenic differentiation, ALP expression levels were used to systematically assess the effect of nanofibrous membranes on the efficacy of early osteogenic induction in BMSCs (Ren et al., 2021). ALP staining (Figures 4A,B) and quantitative analysis of ALP activity (Figures 4D,E) demonstrated that following a 7-day and 14-day culture period, cells in all groups showed the phenomenon of violet-blue precipitate deposition, with the PCZ group showing the densest staining areas and the deepest staining intensity due to synergistic effect of ZIF-8 NPs. The PC group demonstrated significantly higher staining intensity and activity at 14 days (*P* < 0.01) in comparison to the P group. However, no statistical difference was observed between the two groups at 7 days (*P* > 0.05). Quantitative analysis further demonstrated that ALP activity in the PCZ group increased in a time-dependent manner, and its 14-day activity value was elevated approximately 1.6-fold in comparison with that of the P group (*P* < 0.001). The results indicated that ZIF-8 NPs significantly strengthened the early osteogenic differentiation ability of PCZ composite fibrous membranes by regulating osteogenesis-related signaling pathways.

The capacity of the extracellular matrix to mineralize was systematically assessed by means of ARS calcium nodule staining. This is a key morphological marker of advanced osteogenic differentiation (Sculean et al., 2008). Following a 21-day incubation period, the presence of orange mineralized nodule deposition was observed in all groups (Figure 4C). The PCZ group demonstrated the highest mineralization density and strongest staining intensity, followed by the PC group. In contrast, the P group exhibited the lightest staining and the lowest number of calcium nodules. The results of the semi-quantitative analysis conducted by ARS (Figure 4F) demonstrated a consistent trend with staining, with significantly higher OD values observed in the PCZ group compared to the PC group (*P* < 0.01) and the P group (*P* < 0.001). The results obtained above demonstrate that the prepared PCZ composite fiber membrane has the capacity to promote extracellular matrix mineralization and calcium deposition.

### 3.5 *In vitro* antibacterial ability

In the aftermath of GBR, the regenerative outcome of hard and soft tissues is subject to the influence of bacterial colonization and infection (Wang et al., 2020). The introduction of antimicrobial components has been demonstrated to be efficacious in the prevention of infection, as well as in the reduction of inflammation (Brown et al., 2018). In the context of bacterial research, *E. coli* (Gram-negative bacteria) and *S. aureus* (Gram-positive bacteria) have been selected as models to assess the antimicrobial capacity of PCZ nanofiber membranes.

**FIGURE 5:**
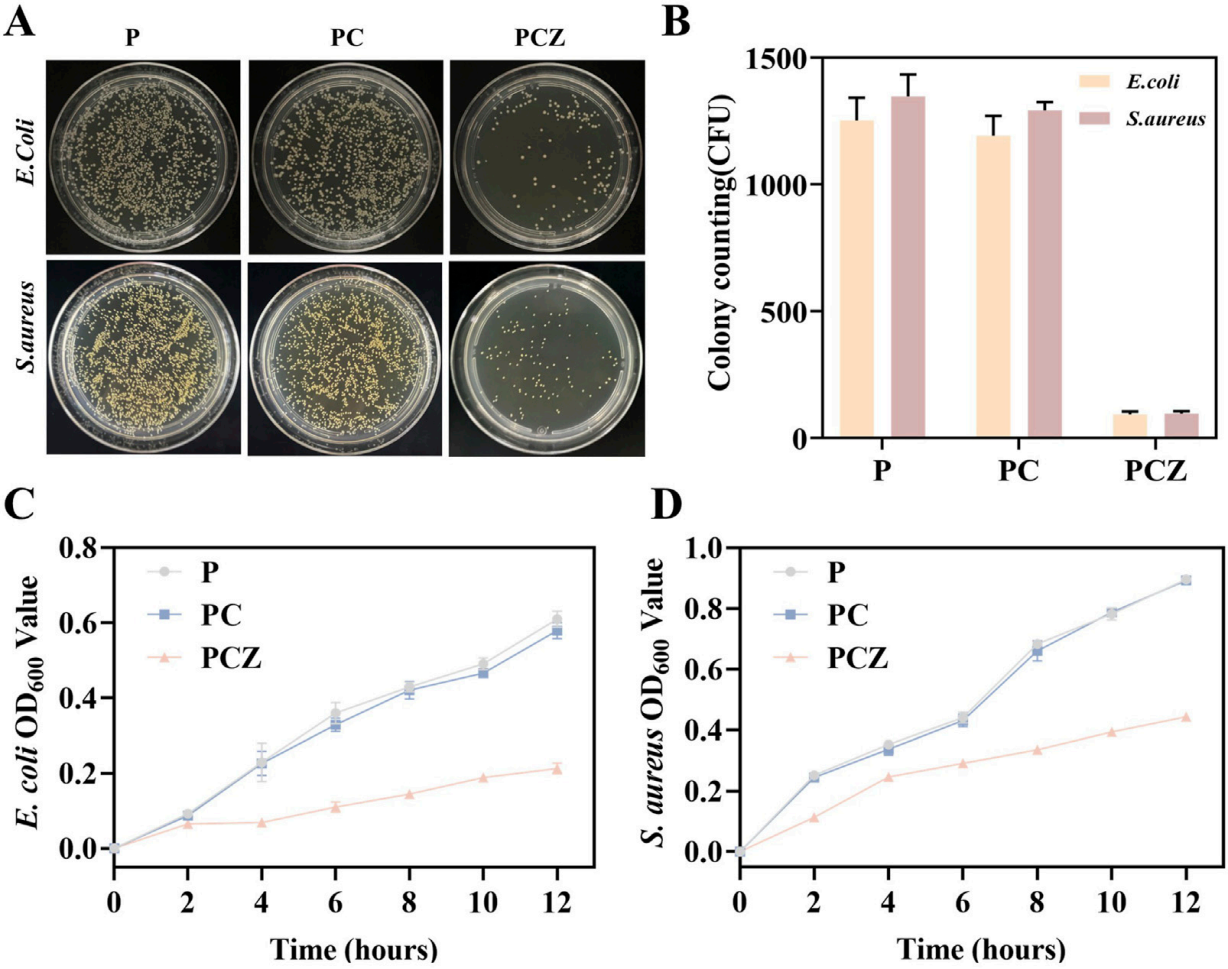
Antibacterial ability of the nanofibers *in vitro*. **(A)** Agar plate images for *E. coli* and *S. aureus* treated with the different membranes. **(B)** Colony counts for *E. coli* and *S. aureus*. OD values of bacterial solution for **(C)** *E. coli* and **(D)** *S. aureus* cocultured with different membranes. Data are presented as mean ± SD (n = 3, *P < 0.05, **P < 0.01, ***P < 0.001).

As demonstrated in Figure 5A, the antibacterial properties of distinct fiber membranes were examined through agar plate counting. In comparison to P and PC, PCZ nanofiber membrane exhibited substantial bacterial inhibition, evidenced by a substantial decrease in the number of bacterial colonies following 12 h of inoculation with *E. coli* and *S. aureus* (Figure 5B). Moreover, OD values of bacterial strains co-cultured with the fiber membranes were investigated. As depicted in Figures 5C,D, after 12 h of co-incubation, the OD value of the P showed no significant difference compared to that of the PC, indicating that Col contributes minimally to the antibacterial activity of the PC membrane. In contrast, the OD values for *E. coli* and *S. aureus* in the PCZ were substantially lower than those in the P and PC. This demonstrates a significantly enhanced antibacterial effect, further confirming that the Zn^2+^ released from the PCZ fiber membrane demonstrates certain inhibitory activity against *E. coli* and *S. aureus*.

Membrane materials have been shown to carry a high risk of infection during the initial week following implantation. To prevent further bacterial colonization, it is essential to ensure a sustained release of Zn^2+^ (Xue et al., 2014). ZIF-8 nanoparticles, derived from PCZ nanofiber membranes, have been demonstrated to be effective in preventing bacterial colonization, with a sustained release capacity of at least 7 days (Figure 2F). This property is crucial in the early stages of bacterial colonization, where it can play a pivotal role in preventing the establishment of bacterial communities (Wu, B. et al., 2019).

### 3.6 *In vivo* bone regeneration

**FIGURE 6:**
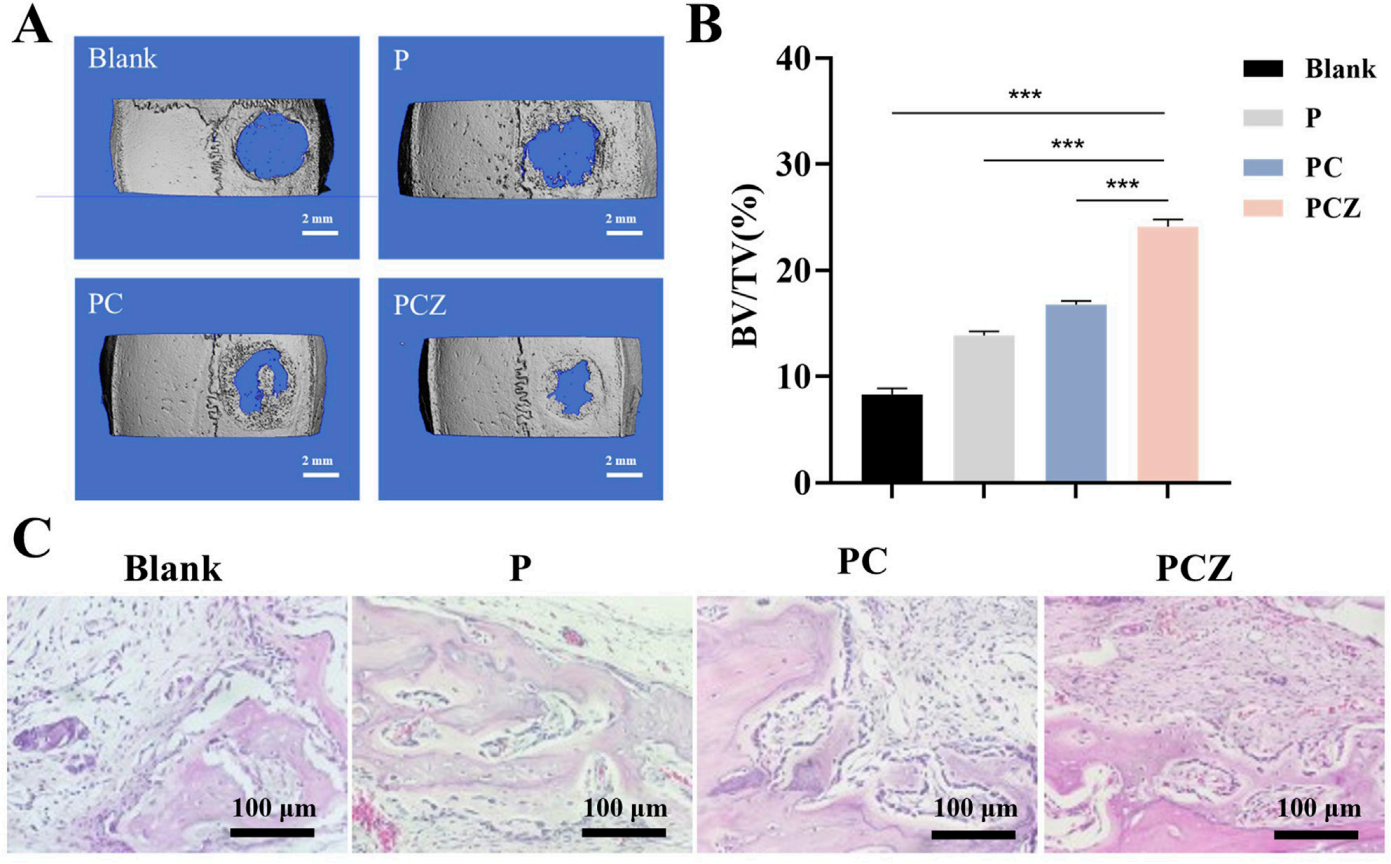
New bone formation in a rat cranial defect model after implantation of the nanofibers. **(A)** Micro-CT reconstructions of the defects after 4 weeks post-surgery in different groups (Blank, P, PC, and PCZ). **(B)** Bone volume to tissue volume (BV/TV) in the calvarial defect determined by micro-CT. **(C)** H&E staining images of sections of different groups after 4 weeks post-surgery. Data are presented as mean ± SD (n = 5, *P < 0.05, **P < 0.01, ***P < 0.001).

The study evaluated the efficacy of PCZ nanofiber membranes on bone tissue regeneration in a rat cranial defect model (diameter: 5 mm). Three types of membrane materials, P, PC and PCZ membranes, were tested in parallel and the results were compared with a “no membrane control” group (Figure 6A). The rats were euthanized 4 weeks after surgery, and the cranial defects were collected and assessed for bone regeneration by Micro-CT and histological analysis.

A reconstructed Micro-CT scan image is presented in Figure 6A, showing newly formed bone within the defect at 4 weeks postoperatively. The 3D reconstructed image demonstrated a significant increase in bone regeneration in the defect treated with PCZ fibrous membrane. Specifically, a significant increase in bone volume to tissue volume (BV/TV) was observed in the PCZ fiber membrane treated site compared to the untreated site and the other fiber membrane treated sites (*P* < 0.01) (Figure 6B).

Moreover, the results of H&E staining further validated the results of the Micro-CT analysis. As demonstrated in Figure 6C, in the blank control group, connective tissue occupied the majority of the bone defect area, with only a minimal amount of new bone formation observable at the edges. Observations revealed the presence of new bone formation on the inner face of the fibrous membrane material in all three groups (P, PC and PCZ). The study demonstrated that the osteoconductive ability of the membrane was indicated by the growth of new bone from the edge of the defect. A significant number of osteoblasts could be seen surrounding the bone defect area in the PCZ group, indicating a promising bone regeneration ability. Furthermore, new bone was discovered beneath the membrane, predominantly originating from its base. This phenomenon can be attributed to the presence of stem cells beneath the dura matter, a characteristic commonly observed in models of cranial defects. For histological assessment of visceral organs, examination of critical organs (heart, liver, spleen, lungs, and kidneys) via H&E staining revealed no significant toxicity or injury (Supplementary Figure S5A). Additionally, in the blood biochemical tests, no abnormalities were detected in WBC, RBC, HGB, PLT, AST, ALT, UREA, or CREA at day 3 (Supplementary Figure S5B). The implanted membranes demonstrated neither hematological nor organ toxicity, providing critical evidence supporting their biological applications. This study has limitations in detecting inflammatory indicators; future research must further examine the pathological manifestations of inflammatory responses.

While this study confirms the macroscopic efficacy of composite materials in promoting bone regeneration through multi-scale characterization, the following limitations must be acknowledged. First, the molecular regulatory mechanisms by which the material mediates bone repair remain incompletely elucidated. Second, although electrospinning technology successfully constructed a biomimetic nanofiber scaffold, scalable production remains constrained by the low throughput of single-nozzle spinning and its sensitivity to processing parameters. Furthermore, this study focused on the early stage of bone repair, primarily monitoring the 7-day Zn^2+^ release kinetics, but the risk of Zn^2+^ accumulation during the later degradation phase and its coupling effect with new bone remodeling rates require further validation through long-term experiments. To address these issues, subsequent investigations will build upon previous signal transduction pathway research to examine whether canonical osteogenic signaling pathways participate in PCZ-induced BMSCs osteogenic differentiation. Simultaneously, the *in vivo* observation period will be prolonged to 12 weeks to further evaluate the spatiotemporal matching between material degradation and bone functional reconstruction, thereby facilitating the material’s advancement toward clinical translation.

## 4 Conclusion

In this study, a novel Col and ZIF-8 reinforced PLGA fiber-guided bone regeneration membrane was successfully prepared by electrospinning. The incorporation of Col into the PLGA-based membranes resulted in a substantial enhancement of their mechanical strength, while the addition of ZIF-8 nanoparticles facilitated the sustained release of zinc ions from the fibrous membranes. The incorporation of ZIF-8 and Col enhanced the cytocompatibility of PLGA membranes to BMSCs; notably, doped ZIF-8 considerably promoted osteogenic differentiation of BMSCs. PCZ fibrous membranes have been demonstrated to provide a basic microenvironment for osteoblasts and have been shown to promote bone defect repair in a rat cranial defect model. Therefore, it is considered that electrostatically spun PCZ fiber membranes are potential candidates for guided bone tissue regeneration applications.

## Data Availability

The raw data supporting the conclusions of this article will be made available by the authors, without undue reservation.
