# Supplementary material for: Multifunctional PLGA/collagen/zeolitic imidazolate framework-8 composite nanofibrous membranes for guided bone regeneration
Source: Front Bioeng Biotechnol. 2025 Jun 27;13:1611948. doi: 10.3389/fbioe.2025.1611948 (PMC12245843; doi:10.3389/fbioe.2025.1611948)
Supplement: Supplementary file 1 [file DataSheet1.docx]

Supplementary Material

# Methods

**Preparation of ZIF-8 nanoparticles**

ZIF-8 nanoparticles (ZIF-8 NPs) was synthesized at room temperature by the water phase method as reported (Pan, Y. et al., 2011). More specifically, First, 1.17g of Zn (NO3)_2_·6H2O was dissolved in 8g of deionized water and 22.70g 2-methimidazole was dissolved in 80g of deionized water. The zinc nitrate solution was mixed with 2-methimidazole solution under stirring at room temperature. After stirring for 5 min, the mixed solution was centrifuged to collect ZIF-8 crystals and washed with DI water for several times. The product was dried at 60℃ in a drying oven overnight. The diffraction pattern of ZIF-8 NPs was detected using an X-ray diffraction (XRD, X’PERT, Panalytical, Holland). The chemical compositions of the ZIF-8 NPs were established by Fourier transform infrared spectrometer (FTIR, Nicolet iS5, Thermo, USA). The morphology of ZIF-8 NPs was obtained by transmission electron microscopy (TEM, Tecnai F20, FEI, USA).

# Results

## Characterization of ZIF-8 nanoparticles

## The ZIF-8 NPs exhibited a characteristic dodecahedral morphology, with a particle size distribution of 120.40 ± 11.83 nm (Supplementary Figure 1A-B). EDS analysis demonstrated a uniform distribution of elements within each of the elements of C, N, O, and Zn (Supplementary Figure 1C). The XRD pattern demonstrated that the synthesized ZIF-8 NPs exhibited identical diffraction peak positions to the simulated crystals, and the diffraction peaks were of high intensity, indicating that the synthesized ZIF-8 NPs were pure phases with high crystallinity (Supplementary Figure 2A). FTIR analysis was conducted to ascertain the peak positions. The absorption peaks at 3134 cm^-1^ and 2930 cm^-1^ correspond to the C-H stretching vibration peaks on the imidazole ring and methyl group, respectively. The absorption peak at 420 cm^-1^ in the IR spectrum corresponds to the Zn-N bond, indicating the formation of Zn and N coordination. The absorption peak at 1583 cm^-1^ is the stretching vibration of the C=N bond on the imidazole ring. The analytical results of these features are in agreement with the results of the literature ( Ordoñez, M. J. C. et al., 2011), indicating the chemical groups in the typical ZIF-8 structure (Supplementary Figure 2B).

**
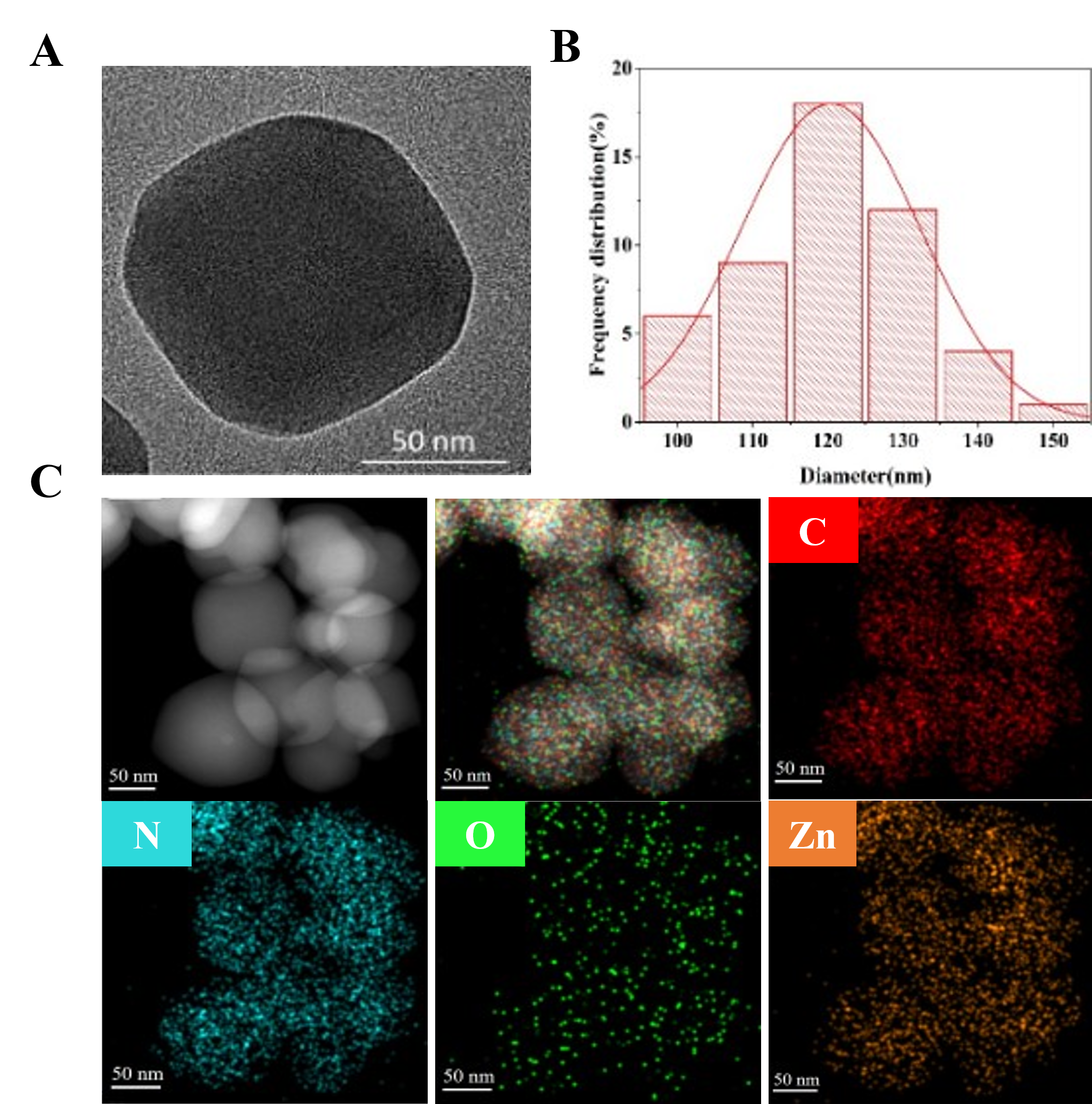
**

**Supplementary Figure 1.** TEM characterization of ZIF-8. (A) TEM images of ZIF-8. (B) Size distribution of the ZIF-8. (C) TEM-mapping images of ZIF-8.

**
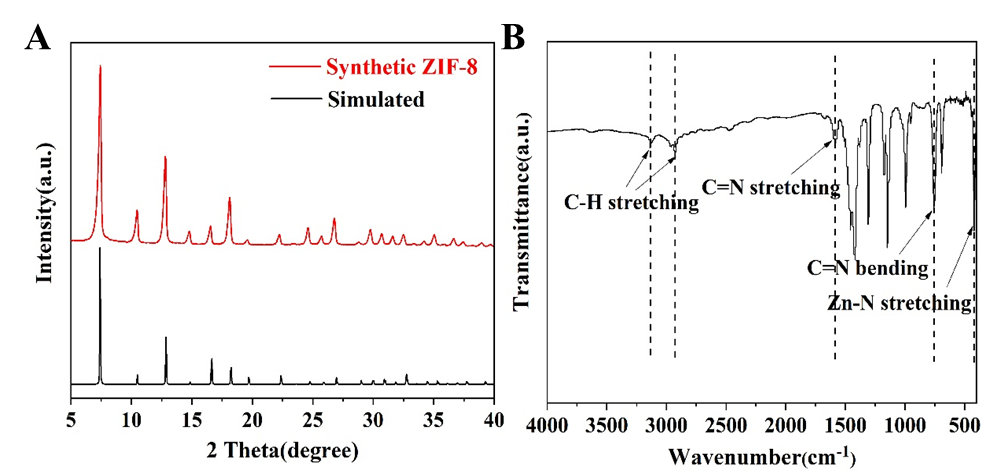
**

**Supplementary Figure 2.** Chemical characterization of ZIF-8. (A) The XRD images of the ZIF-8. (B) The FTIR images of the ZIF-8.

**
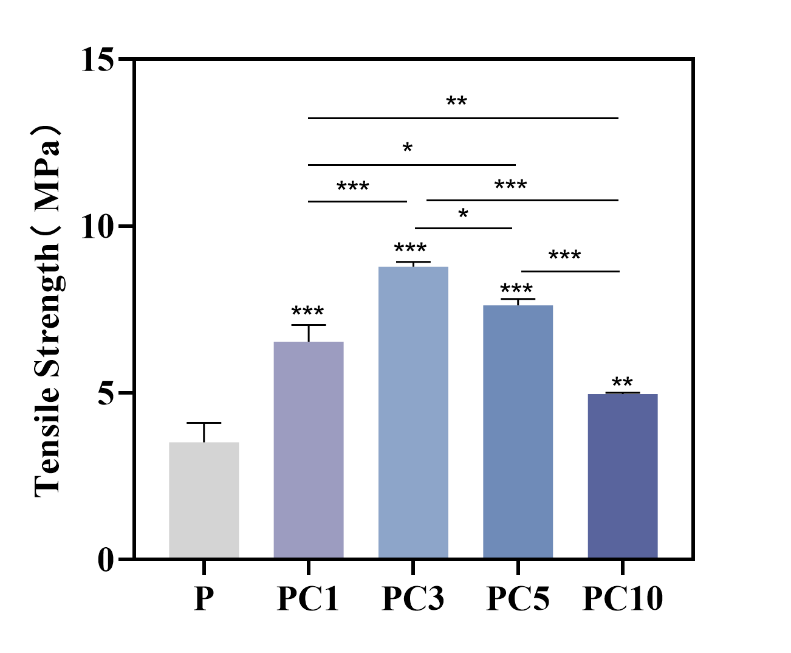
**

**Supplementary Figure 3.** Tensile mechanical properties of different ratios of PLGA/Col. (n = 3, **P* < 0.05, ***P* < 0.01, ****P* < 0.001).


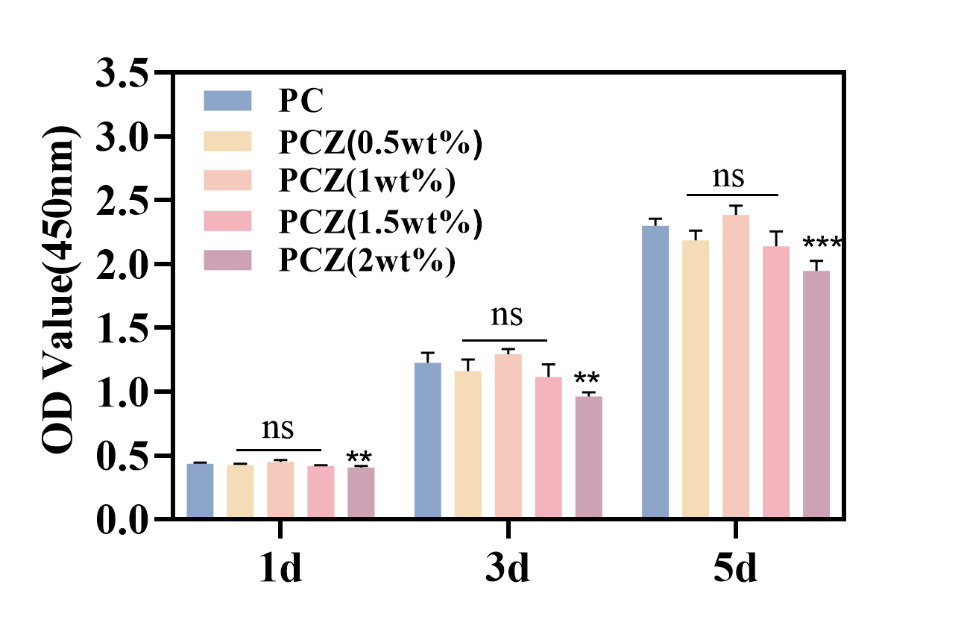


**Supplementary Figure 4.** BMSCs on PC nanofibers and PCZ nanofibers with different concentrations of ZIF-8 NPs. (n = 4, **P* < 0.05, ***P* < 0.01, ****P* < 0.001).


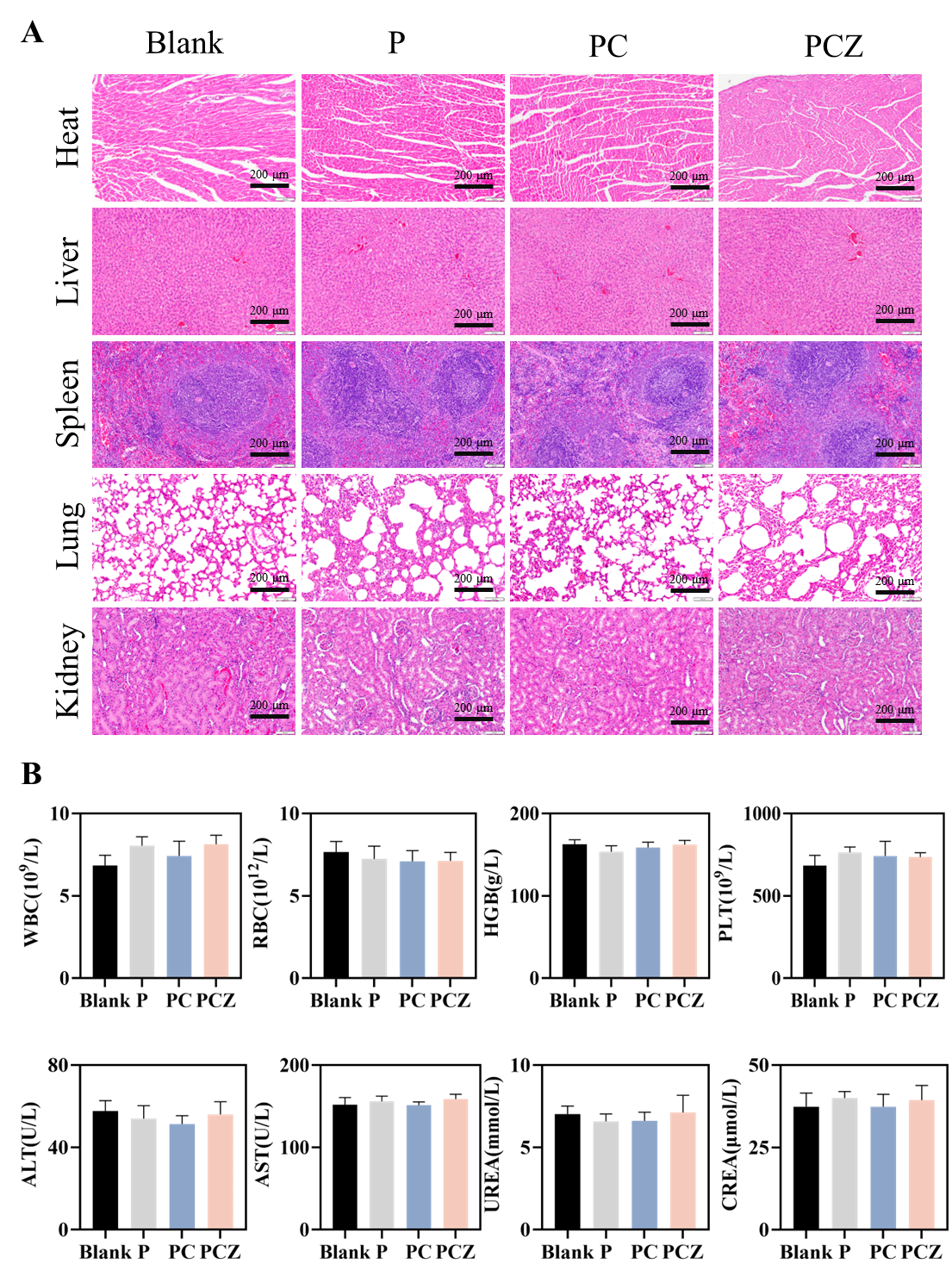


**Supplementary Figure 5.** (**A**) H&E staining assessments of rat vital organs including hearts, livers, spleens, lung and kidneys. (B) Hematological analysis of rat peripheral blood at day 3. Data are presented as mean ± SD (n=5).
